# Chemerin Signaling in Cancer

**DOI:** 10.3390/cancers12113085

**Published:** 2020-10-22

**Authors:** Oliver Treeck, Christa Buechler

**Affiliations:** 1 Department of Gynecology and Obstetrics I, University Medical Center Regensburg, 93053 Regensburg, Germany; 2 Department of Internal Medicine I, University Medical Center Regensburg, 93053 Regensburg, Germany; christa.buechler@klinik.uni-regensburg.de

**Keywords:** cancer, adipokine, chemerin, CMKLR1, signal transduction, survival

The multifunctional adipokine chemerin exerts key functions in inflammation, adipogenesis and glucose homeostasis. Increasing evidence suggests that this adipokine also plays an important role in cancer [1,2,3]. Chemerin signaling in cancer is pleiotropic and able to act on at least three levels: (1) the intracellular signaling of tumor cells, (2) recruiting innate immune defenses to the tumor and (3) the activation of endothelial angiogenesis [2,3]. Recent studies suggested that chemerin exerts both anti-tumoral or tumor-promoting effects, which depend both on its mode of action and the tumor type [2,3]. Whereas leucocyte recruitment by chemoattractant chemerin clearly leads to suppression of tumor growth, the new formation of blood vessels triggered by this adipokine is an important reason for its tumor-promoting function. However, the chemerin-induced intracellular signaling of tumor cells is only beginning to be understood and is able to activate both tumor-promoting and suppressing pathways [2,3].

Chemerin signaling is mediated by its binding to the receptors, chemokine-like receptor 1 (CMKLR1) and G-protein coupled receptor 1 (GPR1). C-C chemokine receptor-like 2 (CCRL2) is a non-signaling receptor proposed to present chemerin at the cell surface [4]. Chemerin receptor mRNA is expressed ubiquitously, but at different levels in normal tissues, and is up- or down-regulated in cancer in a tissue-specific manner [1,2,3]. Regarding receptor protein expression, there is a profound inconsistency with the mRNA data, which has to be resolved in future studies. 

With regard to the effect of chemerin and its receptors on cancer survival, tumor-specific differences have been reported. Whereas in many cancer entities, such as breast cancer, ovarian cancer, melanoma, hepatocellular carcinoma (HCC), adrenocortical carcinoma (ACC) and acute myeloid leukemia (AML), higher levels of serum chemerin and/or high expression of different sets of chemerin receptors were associated with increased survival, in other cancer types, such as gastric cancer, high serum levels of chemerin and high expression of CMKLR1 and GPR1 were reported to associate with decreased survival. In Non-Small-Cell Lung Cancer (NSCLC), chemerin was downregulated in the cancer tissues [5,6]. Moreover, serum chemerin is of diagnostic and prognostic value in NSCLC [7,8]. Of note, serum chemerin was even induced in the cancer patients [7,8]. Thus, locally produced chemerin may have effects distinct from systemic chemerin [2].

Chemerin is secreted as an inactive protein and is activated by C-terminal processing. Proteolysis produces at least three different active chemerin variants. However, most studies so far measured total chemerin levels with pan-ELISAs without distinguishing the different active and inactive chemerin isoforms. Effects of the most active variants were mainly tested with regard to chemotaxis. However, these isoforms may also affect angiogenesis or might play so far unknown roles in cancer. Detailed characterization of the chemerin variants by, e.g., mass spectrometry and measurement of chemerin activity by, e.g., Tango assays is essential to further reveal the multiple effects of this adipokine on cancer development [1,9].

Moreover, serum chemerin is positively correlated with hypertension [10], which is a common comorbidity in cancer patients [11]. This has to be taken into account when examining chemerin levels in cancer. A recent meta-analysis including 12 studies (876 cases and 739 healthy controls) showed a pooled standardized mean difference of 1.47 (which is the size of the effect in each study relative to the variability in that study) for chemerin in patients with cancer and controls [12]. The limitation of the studies was that cancer patients and control groups were most often not matched for body mass index [12]. Chemerin is well known to be increased in obesity [13], and a higher body mass index and less cases with comorbidities in the controls have to be considered.

Various downstream pathways are activated by chemerin. Chemerin increases intracellular calcium and decreases cyclic AMP levels. Chemerin can also induce the phosphorylation of p42-p44 MAP kinases [1,14]. The binding of chemerin recruits β-arrestin 1 and 2 to CMKLR1 or GPR1 [1]. These scaffolding proteins influence the signaling duration and strength of several receptors and are involved in tumor development. While β-arrestin 1 seems to mostly promote tumor growth, β-arrestin 2 prevents cancer growth and angiogenesis [15]. The transcription factor serum-response factor (SRF) has important roles in cancer progression. Chemerin activates SRF and its downstream target early growth response-1 (EGR1). EGR1 is regarded a tumor suppressor, and inhibits cell proliferation and induces apoptotic cell death [16]. C-Fos is a further downstream target of SRF and may act as an oncogene or as a tumor-suppressor. Chemerin also destabilizes the CMKLR1/phosphatase and tensin homolog (PTEN) complex in hepatocytes and thereby enhances activity. This activity of chemerin suppresses hepatocellular carcinoma (HCC) metastasis through decreasing p-Akt levels [14]. Prostate and sarcoma cancer cell lines exposed to chemerin had higher PTEN activity. Programmed death ligand 1 (PD-L1) is commonly upregulated on tumors and blocks the activity of T-cells. As a consequence of PTEN reactivation, PD-L1 was suppressed and T-cell cytotoxicity was enhanced by chemerin [17]. Both of these studies described higher PTEN protein levels upon chemerin overexpression or supplementation of exogenous chemerin [14,17]. In prostate cells, PTEN mRNA was also induced [17]. Whether chemerin upregulates PTEN expression and in addition destabilized the CMKLR1/PTEN complex in both tumor cell types needs further studies. Xenograft mouse models revealed that chemerin decreases the levels of phosphorylated p38 MAPK and β-catenin in adrenocortical cancer. The phosphorylation of p38 and ERK1/2 MAP kinases was elevated by chemerin in gastric cancer cells. Given that MAPK pathways enhance tumor growth and metastasis in different cancer types, several other studies support the finding that chemerin promotes tumor growth via these mechanisms. Chemerin-triggered activation of matrix metalloproteinases (MMP) has been reported in a variety of studies and may contribute to the invasion and migration of cancer cells. In breast cancer, chemerin nevertheless reduced MMP-2 and MMP-9 secretion. Chemerin inhibited tumor growth in breast-cancer-cell-injected mice and protected against breast-cancer-induced bone loss [18]. Very little is known about the role of chemerin in non-solid tumors. In acute myeloid leukemia, chemerin was downregulated and was an independent prognostic factor [19].

In conclusion, with regard to chemerin signaling in different tumor types, convincing evidence for both the anti-tumoral and tumor-promoting effects of this adipokine exist. Chemerin and its receptors are promising targets for the treatment of different cancers, and more effort is needed to finally resolve the multiple pathways downstream of CMKLR1 and GPR1. 
